# Supplementary material for: Fabrication of PEDOT:PSS/Ag2Se Nanowires for Polymer-Based Thermoelectric Applications
Source: Polymers (Basel). 2020 Dec 8;12(12):2932. doi: 10.3390/polym12122932 (PMC7764283; doi:10.3390/polym12122932)
Supplement: Supplementary file 1 [file polymers-12-02932-s001.pdf]

Supplementary Information:

Fabrication of conductive polymer/inorganic nanoparticles composite films: using PEDOT:PSS with Ag_2Se nanowire for polymer-based thermoelectric applications

Dabin Park, Hyun Ju, Minsu Kim and Jooheon Kim*

School of Chemical Engineering & Materials Science,

Chung-Ang University, Seoul 06974, Korea

*Corresponding author: jooheonkim@cau.ac.kr (J. Kim)

Supporting Information Contents:

1. Figures

2. Tables

1. Figures

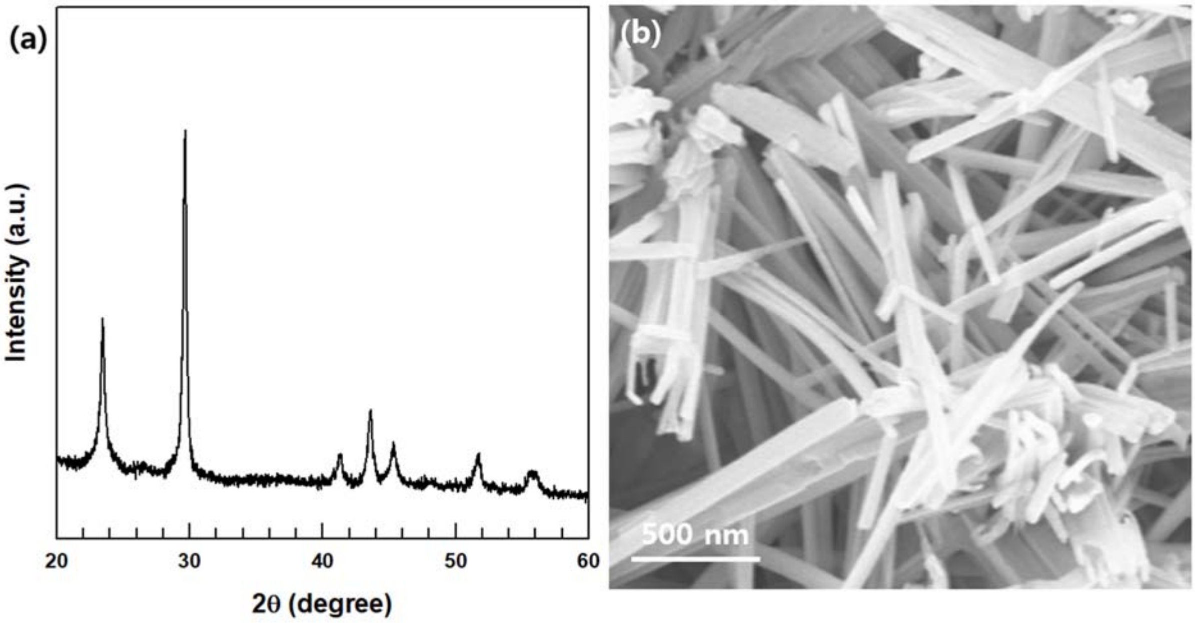

Figure S1. (a) XRD pattern and (b) FE-SEM images of synthesized Se NWs

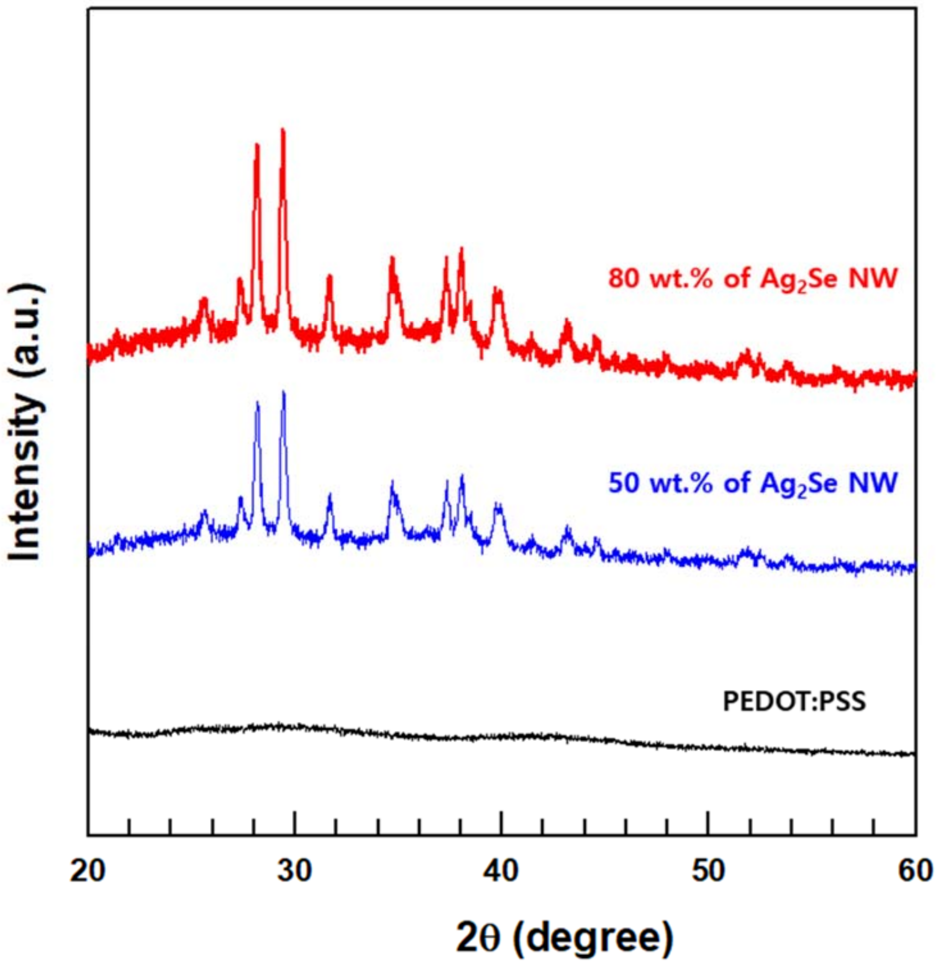

Figure S2. XRD pattern and Pristine PEDOT:PSS, Ag_2Se NW/PEDOT:PSS with 50 and 80 wt.% of Ag_2Se NWs

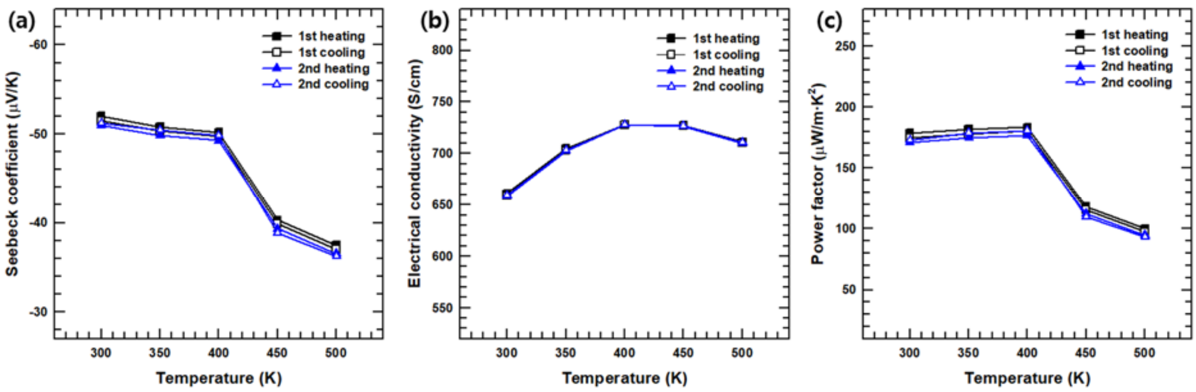

Figure S3. Temperature-dependent (a) Seebeck coefficient, (b) electrical conductivity, and (c) power factor values of PEDOT:PSS composite films with 80 wt.% of Ag_2Se during multiple heating and cooling cycles.

2. Tables

	σ_s (S/cm)	σ_p (S/cm)	S_s (μV/K)	S_p (μV/K)	κ_s (W/m·K)	κ_p (W/m·K)
In this study	581	788.6	-145	12.8	0.53	0.22
Previously reported	~497	~700	~-140	~16	~0.6	~0.29
References	Ding *et al.*[34]	Luo *et al.*[25]	Ding *et al.*[34]	Luo *et al.*[25]	Zhao *et al.*[18]	Kim *et al.*[24]

Table S1. Measured electrical conductivity, Seebeck coefficient, and thermal conductivity for the calculation of the series- and parallel-connected models.
